# Isolated Vaginal Neurofibroma Presenting as Vaginal Wall Cyst: A Rare Case Report With Review of Literature 

**Published:** 2016-03

**Authors:** Sarita Nibhoria, Kanwardeep Kaur Tiwana, Manmeet Kaur, Richa Phutela

**Affiliations:** Department of Pathology, Guru Gobind Singh Medical College AND Hospital, Faridkot, Punjab, India

**Keywords:** Vaginal Cyst, Neurofibroma, Histopathology

## Abstract

Neurofibromas commonly involve peripheral nervous system. Isolated neurofibroma of vagina is very rare tumor and usually associated with Von Recklinghausen’s disease. Vulva is the most frequent location of neurofibroma of genital tract followed by clitoris and labia. We present a rare case of neurofibroma of vaginal wall presented as vaginal cyst in a 52 year old female with no history of any other symptoms related to Recklinghausen’s disease. Excision biopsy was done and on the histopathological examination non-encapsulated, well circumscribed mass composed of spindle shaped cells with wavy nuclei and bland nuclear chromatin was noted. Immunohistochemistry revealed strong positivity with S-100.

## Introduction

Isolated neurofibroma of female genital tract is very uncommon and mostly associated with neurofibromatosis. Neurofibroma as such is a disease of peripheral nervous system and occurs most commonly in the extremities. Amongst female gentital tract, neurofibroma involves most commonly vulva, clitoris and labia but rarely be seen in vagina, cervix, endometrium, myometrium as well as urinary tract (1). Neurofibromas are categorized as cutaneous neurofibromas, intraneuralneurofibromas, massive soft tissue neurofibromas and sporadic neurofibromas or those associated with neurofibromatosis-1 (2, 3). The solitary lesions are rare and usually they are not associated with any systemic symptom. We present a rare case of isolated neurofibroma of vaginal wall in 52 year old female presenting as vaginal cyst.

## Case report

A 52 year old female patient (P2L2) presented in gynaecology department of a tertiary care center at Faridkot, with a history of painless swelling in vagina for 2 months. On examination mass was protruding from lateral wall of vagina, no ulceration, redness and discharge was noted. Clitoris was healthy. Rest of the external genitalia was also healthy. There was no other mass or swelling in the body. There was no skin abnormality. This patient did not have any features of Von Recklinhausen’s disease.

**Figure 1:**
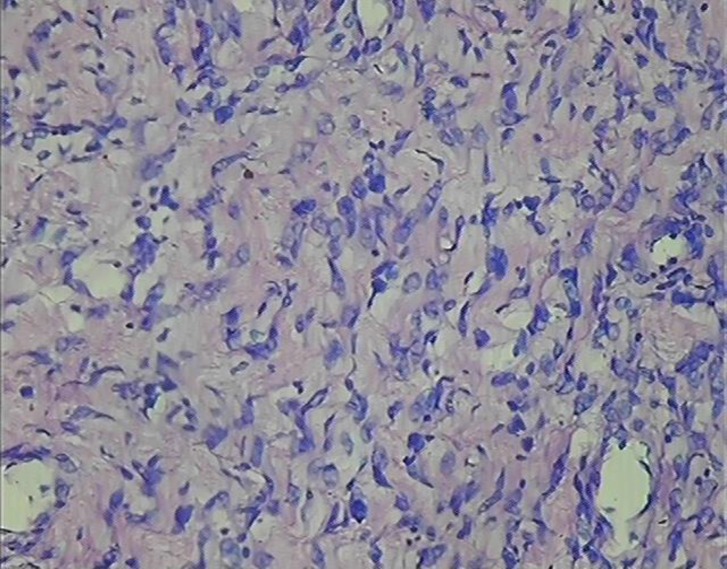
(400x, H&E Stain) Microphotograph showing wavy nuclei characteristic of neurofibroma

Excision biopsy of the lesion was done and sent to pathology department for histopathological examination. On gross examination one grey white soft tissue piece measuring 1×1×1cm was noted. Light microscopic examination revealed normal appearing epidermis, underlying subepithelium shows non-encapsulated tumor composed of a mixed proliferation of spindle shaped cells with wavy nuclei and bland nuclear chromatin. No atypia or necrosis was seen in the sections examined (Figure 1). A diagnosis of neurofibroma was made on microscopic examination which was confirmed on immunohistochemistry. On Immunohistochemistry strong positivity for S-100 was noted. Patient was followed-up for 6 months for any signs of recurrence and other disability but no recurrence or disability found.

## Discussion

Neurofibroma of genital tract is very uncommon. Amongst them vulva is the most frequent location of neurofibroma followed by clitoris and labia but rarely found in vagina, cervix, endometrium, myometrium as well as urinary tract (1). Till date, literature on isolated vaginal neurofibroma is scarce.

Mourali et al (4) and Yayli et al (5) reported a 71 year old patient with vaginal neurofibroma with Von Recklinghausen disease. Our patient was at 52 years old and hasn’t had Von Recklinghausen disease.

Baulies et al reported a 20-year-old woman with a history of type-1 neurofibromatosis with a vaginal nodule neurofibroma (6). But our patient did not have history of type-1 neurofibromatosis.

Sharma et al reported huge localized vaginal neurofibromatosis as an unusual cause of postmenopausal bleeding (7). In our report, the bleeding wasnot a manifestation.

Eusebi and Schönauer reported Pigmented vaginal neurofibroma. In our report there were no pigmentations (8).

Gold published about Neurofibromatosis of the bladder and vagina. In our report the vagina only was involved (9).

Drescher and Herzog published about neurofibromatosis of the vulva and vagina. In our report only vagina was involved (10).

Marmey and Lacroix published about Recklinghausen disease and pregnancy (11). But our patient was not pregnant at presentation and the neurofibroma was solitary.

Belvederi et al reported Anatomo-clinical findings on a case of neurofibroma of the vagina (12).

De Jorio and Belfiore (13) reported rare case of vaginal localization in the course of Recklinghausen's disease. Stingl published about contribution to the knowledge of primary neurofibroma of the vagina (14).

Norris and Cooper reported about Primary neurofibroma of the vagina (15).

Benign solid tumors of vagina like leiomyoma, condylomaaccuminata should be kept in mind. On histopathological examination no features of malignancy were observed, hence excision proved to be therapeutic (16). Patients with excision should be followed-up for recurrence and any other abnormality.

## Conclusion

To conclude neurofibroma of female genital tract is not common, amongst that vaginal neurofibroma is rare. Systemic evaluation for the presence of café-au-lait spots or any other swelling should be performed to rule out Von Recklinghausen’s disease. Isolated vaginal neurofibroma is rare and should be kept in differential diagnosis of benign solid tumors of vagina.
